# Effects of information, education, and communication campaign on a community-based health insurance scheme in Burkina Faso

**DOI:** 10.3402/gha.v6i0.20791

**Published:** 2013-12-06

**Authors:** Patience Cofie, Manuela De Allegri, Bocar Kouyaté, Rainer Sauerborn

**Affiliations:** 1 PATH Ghana, Cantonments, Accra, Ghana; 2 Department of Tropical Hygiene and Public Health, Graduate School of International Public Health, Heidelberg University, Heidelberg, Germany; 3 Ministry of Health, Ouagadougou, Burkina Faso

**Keywords:** community-based, health insurance, IEC strategies, health promotion

## Abstract

**Objective:**

The study analysed the effect of Information, Education, and Communication (IEC) campaign activities on the adoption of a community-based health insurance (CHI) scheme in Nouna, Burkina Faso. It also identified the factors that enhanced or limited the campaign's effectiveness.

**Design:**

Complementary data collection approaches were used. A survey was conducted with 250 randomly selected household heads, followed by in-depth interviews with 22 purposively selected community leaders, group discussions with the project management team, and field observations. Bivariate analysis and multivariate logistic regression models were used to assess the association between household exposure to campaign and acquisition of knowledge as well as household exposure to campaign and enrolment.

**Results:**

The IEC campaign had a positive effect on households’ knowledge about the CHI and to a lesser extent on household enrolment in the scheme. The effectiveness of the IEC strategy was mainly influenced by: (1) frequent and consistent IEC messages from multiple media channels (mass and interpersonal channels), including the radio, a mobile information van, and CHI team, and (2) community heads’ participation in the CHI scheme promotion. Education was the only significantly influential socio-demographic determinant of knowledge and enrolment among household heads. The relatively low effects of the IEC campaign on CHI enrolment are indicative of other important IEC mediating factors, which should be taken into account in future CHI campaign evaluation.

**Conclusion:**

The study concludes that an IEC campaign is crucial to improving the understanding of the CHI scheme concept, which is an enabler to enrolment, and should be integrated into scheme designs and evaluations.

Community-based health insurance (CHI) schemes are important for providing the poor access to basic healthcare and protection from catastrophic health expenditures (1–6). However, evidence from several studies shows low enrolment in schemes in sub-Saharan Africa (SSA) (3, 5–7). This low participation has partly been attributed to limited use of information, education, and communication (IEC) strategies that drive the implementation and management of the schemes (8–10). IEC provides a platform for the discussion of important health issues to foster an understanding of concepts, underlying principles, and benefits of health initiatives. IEC is essential to achieving better health outcomes in all public health interventions (11–13).

IEC is considered as an imperative in expanding access of the poor to CHI schemes, but it has not been explicitly examined as one of the essential elements in the designs and evaluations of health insurance schemes. In this article, we examine the effect of IEC on enrolment rates in a CHI scheme. We do so by demonstrating the relevance of IEC within CHI scheme promotion in Nouna, Burkina Faso.

## Background

The impact of CHI schemes in developing countries has attracted significant attention in recent times, and it is argued to have huge potential to contribute significantly to achieving the millennium development goals (14, 15). With the exception of Ghana where coverage of CHI is relatively high, enrolment levels have remained low in the rest of sub-Saharan African countries, thus raising concerns about sustainability of such schemes. CHIs tend to target all socio-economic groups including rural and urban resource poor, and marginalised, thus making their sustainability largely dependent on high enrolment rates (7, 16).

Factors limiting high enrolment in CHIs include misconceptions and the lack of consumers’ understanding about the concept, underlying principles, and the benefits, as noted in earlier studies in Burkina Faso, Senegal, and Ghana (8–10). The misconceptions about the schemes reflect the ineffective sensitisation and marketing of schemes among potential clients and other essential stakeholders as noted in Ghana and Senegal (9, 10).

IEC has been extensively used to improve immunisation coverage, promote sexual and reproductive health, family planning, reduce fertility levels, and increase uptake of insecticide-treated nets (ITNs) and sulfadoxine–pyrimethamine (SP) for the treatment and control of malaria in African and Asian countries (17–21). It is recognised as a viable and cost-effective approach to addressing broader determinants of health, risk factors, building trust and commitment, fostering community participation, and empowerment towards development and implementation of health initiatives (22–26). The World Health Organization (WHO) and the World Bank recognises the important role of IEC in the achievement of health, nutrition, and population (HNP) goals and recommended its inclusion in health programmes for sustainable and better health outcomes (12, 23).

Notwithstanding IEC's importance, its implementation and evaluation in CHI scheme design and evaluation has received little attention. CHI evaluations have often focused on socio-economic, managerial, and health system issues perceived to underpin the success of schemes. Populations’ understanding of CHI scheme concept is vital for the scheme's immediate success and sustainability.

Since 2004, Burkina Faso has been piloting a district-wide CHI scheme in its Nouna Health District (NHD), with an IEC campaign as a component. The aim is to improve understanding of the CHI concept and benefits, gain social approval for the underlying principles, and enhance enrolment rates in the scheme.

This study sought to assess the effects of the IEC campaign on knowledge and enrolment in the Nouna CHI scheme among households. It also identified the key IEC campaign variables that were associated with the improvement.

The study addressed three questions: (1) To what extent did the IEC campaign enhance households’ understanding of the CHI scheme? (2) To what extent did the IEC campaign influence households’ enrolment in the CHI scheme? (3) Which IEC campaign components were important in enhancing knowledge and enrolment?

This article is expected to contribute to better understanding of the design and implementation of CHI initiatives in SSA by adding to the already known social factors influencing CHI enrolment in the region.

## Method

### The study area

The study was carried out in the NHD, located in the Kossi Province, North-West of Burkina Faso with a total estimated population of 320,232 (27). The district is largely rural with semi-urban characteristics in Nouna, the district capital. The majority of the inhabitants are Muslims with few Christians and traditionalists. There are six major ethnic groups with different dialects namely Mossi, Darfing, Bwaba, Marka, Fulani, and Samagho with Dioula as the lingua-Franca. Three quarters of the population is illiterate and depend mostly on subsistence crop farming and livestock breeding. Radio coverage was relatively high, reaching the entire district, although no radio station was located in the Nouna district at the time of the study. Television coverage, however, was very low in the district, particularly in the rural areas, due to the lack of electrification and low ownership rate of television sets.

The only hospital situated in Nouna had 17 supporting health posts known as Centre de Santé et de Promotion Sociale (CSPS) in 2004, but this increased to 23 in 2010. Each of these CSPS has a local pharmacy managed by the community through health committees known as Comités de Gestion (COGES). The COGES is also responsible for community mobilisation, health education, and health promotion. The district also hosts a Health Demographic Surveillance System (HDSS) at the Centre du Recherche en Santé de Nouna that serves as a database for planning and research in the area (27).

### The CHI scheme

The CHI scheme, known locally as *Assurance Maladie à base Communautaire* (*AMBC*), was progressively introduced in the NHD from 2004 to 2006. It aimed at reducing financial barriers to health services and improving uptake of healthcare services and ultimately the health status of the population. Each year, one-third of the eligible population was targeted. As described elsewhere (5, 8), the Nouna CHI scheme is not-for-profit and voluntary. It was implemented under the guidance of a Steering Committee (SC) consisting of local leaders, representatives from the Centre du Recherche en Santé de Nouna and District Santé de Nouna. In 2004, an IEC campaign was incorporated as a key component of the implementation plan to create high awareness and understanding of the CHI scheme among the target population.

### The Nouna CHI campaign strategy

The CHI campaign was implemented between January and May 2004 in the 15 communities (12 villages and 3 town sectors) that were eligible for the scheme implementation. The primary IEC targets were household heads (formal decision-making authorities within households) living in the 15 communities. The secondary targets were religious leaders, ethnic groups and community heads, heads of government and non-government agencies, and media experts in the district. These were valuable partners in the promotion as they occupied key positions and could affect changes in social norms and behaviours or enforcement of policy changes in their constituencies (26).

The CHI campaign team comprised the scheme management personnel, a social mobilisation officer and research coordinator, a health promotion officer from the District Santé de Nouna, *Animateurs* (community mobilisation and communication agents), and community volunteers (three persons from each community). The community volunteers nominated by their respective community heads (*Délégates*) and the *Animateurs* were given appropriate training in social mobilisation and promotion to enable them support and sustain the campaign effectively.

Social marketing, advocacy, and stakeholder sensitisation seminars formed the core strategy to reach the target groups (28). The CHI campaign was launched with a call by the District Authority on government representatives, civil society, and community heads to support the CHI initiative for massive enrolment in the scheme.

**Fig. 1:**
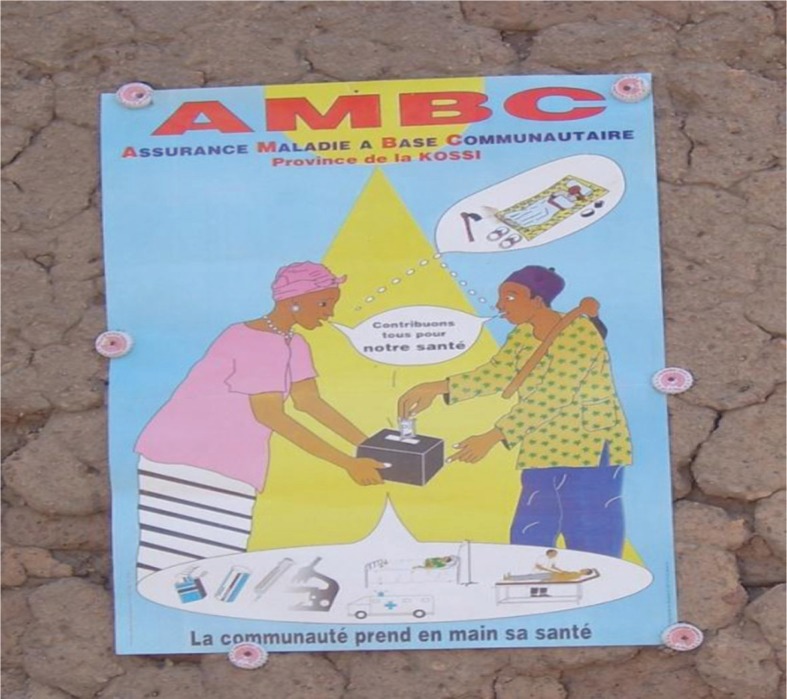
Sample of campaign poster.

Prior to the launch, campaign messages and materials were jointly developed by representatives of the CHI campaign team, the district health promotion unit, the SC, and a local artist, who were pre-tested in two villages and a sector in Nouna Township. Revisions reflected the perceptions, aspirations, and understanding of the target populations. Key campaign messages highlighted the benefits of the health insurance concept *sharing community health burden through financial contributions of community members* (see Fig. 1, CHI Poster), thereby protecting individual households from the risk of catastrophic medical expenditures. Campaign print materials included t-shirts, calendars, and billboards. Posters were pasted at vantage points in all eligible communities, government offices, and health facilities, while billboards were mounted at central areas in Nouna; calendars and t-shirts were distributed to all community, agency, and religious heads as well as individual household heads that enrolled in the scheme.

The above-mentioned materials were complemented with a mixture of mass media and interpersonal channels. Radio was the main mass media channel for the campaign with Radio CEDECOM, broadcasting the CHI campaign message and facilitating roundtable thematic discussions. Alternate 30-min discussion broadcast in Dioula and French twice every week (afternoons and evenings) were aired throughout the campaign period; and from mobile vans with loudspeakers in each village once every week throughout the campaign period to diffuse campaign messages.

The CHI team also organised interactive dialogues – congregational, ethnic, and community group discussions with the assistance of religious and community administrative heads (Délégates). Traditional folk media channels such as the *Griots* (praise singers/poet) were also used in some communities to mobilise people for community discussions.

### Study design and data collection

The study used cross-sectional design and mixed methods that combined quantitative and qualitative data collection methods to complement each other. Data collection took place in July 2004, independent of the well-known longitudinal surveys in the district. The data sources comprised review of CHI documents, the HDSS database, household survey, key informant interviews, and participant observations. The HDSS database provided the sampling frame of 3,125 households from 15 communities. A systematic random sampling method was used to select a representative household sample from the 15 communities. The sample size was based on Cochran's formula for categorical data: with (*α*)= 0.05, thus (95% confidence level), margin of error (*d*) = 10%. Assuming 50% of targeted households had some knowledge about the CHI initiative, this formula yielded a minimum sample size of 93. Assuming a design effect associated with cluster sampling = 2.0, and participant response rate of 72%, the sample size was (93 × 2)/0.72 = 259, and this was rounded up to 260 (29, 30).

Community size was given careful consideration in the selection of households since the households differed in size. The selection process started with the estimation of the overall sample size after which a weighted value was assigned to each cluster (community) based on its size by dividing the total population of the 15 clusters (3)125 by the population of each cluster (31, 32). The weighted value for each cluster was then multiplied by the sample size 260 to obtain the number of households to be selected from each community out of which systematic random sampling was used to select the required number of households from each community.

Qualitative in-depth interviews and a meeting with the CHI project management team were carried out to complement the survey data. Twenty-two key informants in decision-making positions in the district were purposively selected for the in-depth interviews. The participants were selected from a list of about 50 local leaders, heads of government agencies, and non-governmental organisations as well as community *délégates* in the district. These included heads of the local authority, religious leaders, and agency heads (Health, Education, Agriculture, Water and Sanitation, and Animal Husbandry Services). A meeting was also held with four members of the CHI project management team, to ascertain perceptions on the campaign strategy. The assessment themes included CHI concept, the benefit package, implementation arrangements, and factors to improve future campaigns.

### Outcome variables and covariates

The survey assessed household heads or their representatives’ exposure to the campaign, and its relationship to knowledge and enrolment. The key outcome variables of interest in this study were: (1) household knowledge, and (2) enrolment in the CHI scheme. Data were gathered on households’ exposure to CHI campaign messages, and their relationship to knowledge and enrolment in the scheme. Campaign exposure was measured by an unprompted question of whether respondents had heard about the CHI scheme or not. The degree of exposure among those exposed to the CHI campaign messages was further measured by the frequency of households’ access to communication channels (intensity of exposure). An individual who received CHI information weekly and had access to more than two of the campaign channels was considered to have had frequent exposure to the CHI message.

Knowledge was defined as a categorical dichotomous outcome and therefore rated as follows: ‘1’ for adequate knowledge, if the respondent could recall the concept of CHI and by mentioning at least three out of six predetermined areas of the benefit package; ‘0’ if otherwise. The understanding of the CHI concept and community leaders’ participation were viewed as a critical first step towards influencing decisions. Community leader participation was also defined as a dichotomous outcome and coded ‘1’ if the head of a community took an active part in the mobilisation of his constituents and ‘0’ if otherwise. Ownership of radio and television was coded as ‘1’ if the respondent owned a radio set; ‘2’ if the respondent owned a radio and/or television set; ‘0’ if the respondent had neither radio nor television. Enrolment was coded as ‘1’ if the respondent did enrol in the scheme; and ‘0’ if the respondent did not.

### Data analysis

Bivariate analysis and multivariate logistic regression models were used to assess the association between the campaign intervention and (1) household knowledge, and (2) household enrolment. The bivariate analysis was performed to assess the association between relevant variables, and it preceded the multivariate logistic regression analysis. A univariate logistic regression analysis was used to identify the significant individual determinants of knowledge and enrolment for inclusion in the multivariate logistic regression (33). Model 1 in Tables 1 and 2 assessed the effects of IEC variables on the dependent variables, while Model 2 in both tables included selected socio-demographic variables. All significant variables from the bivariate analysis were included in the multivariate logistic regression analysis. In addition, all variables deemed relevant based on literature and professional experience were included in the analysis.

**Table 1:** Selected socio-demographic variables

	Frequency (*N*=250)	
	
Socio-demographic variables	No.	%
Location		
Urban	143	57.2
Rural	107	42.8
Gender		
Male	222	89
Female	28	11
Marital status		
Single (Never married)	11	4.4
Married	220	88
Widow/widower	18	7.2
Divorced	1	0.4
Educational status		
Secondary and above	30	12
Primary school	52	20.8
No school	168	67.2
Occupational status		
Farmer	158	63.2
Salaried workers	21	8.4
Unemployed	20	8
Others	51	20.4
Religious affiliation		
Moslem	142	56.8
Catholics	75	30
Traditionalist	20	8
Protestant	13	5.2
Ethnicity		
Dafing	77	30.8
Bwaba	69	27.6
Mossi	34	13.6
Samagho	33	13.2
Fulani	31	12.4
Others	6	2.4
Radio and television ownership		
Radio	160	64
Television	51	20.4
None	39	15.6

**Table 2:** Multivariate logistic regressions on determinants of CHI knowledge

			Regression results				
				
	Knowledge level		Model 1		Model 2	
			
Variables	Adequate (*n*=150) %	Inadequate (*n*=40) %	OR	CI	OR	CI
IEC variables						
Exposure to channels						
None	11.3	35.0	1.00		1.00	
One	44.0	55.0	1.96	0.77 – 5.01	1.48	0.51–4.26
Two or more	44.7	10.0	8.99**	2.13 – 38.01	10.36**	2.12–50.66
Frequency of information from CHI team						
Less frequent	71.3	92.5	1.00		1.00	
Frequent	28.7	7.5	2.41	0.65 – 8.89	2.63	0.62–11.19
Frequency of information from mass media						
Less frequent	61.3	80.0	1.00		1.00	
Frequent	38.7	20.0	0.84	0.31 – 2.31	0.65	0.21–1.97
Community participation						
No participation	36.0	60.0	1.00		1.00	
Participation	64.0	40.0	1.44	0.64 – 3.28	1.75	0.68–4.51
Ownership of radio						
None	28.7	45.0	1.00		1.00	
Radio	71.3	55.0	1.2	0.56 – 2.58	1.12	0.46–2.75
Socio-demographic characteristics						
Sex						
Female	13.3	5.0			1.00	
Male	86.7	95.0			0.48	0.08–2.73
Residence						
Rural	48.0	32.5			1.00	
Urban	52.0	67.5			0.42*	0.17–1.07
Age in years						
< 36	26.7	20.0			1.00	
36–54	68.0	72.5			0.53	0.18–1.49
≥ 55	5.3	7.5			0.96	0.14–6.65
Marital status						
Never married	4.0	2.5			1.00	
Married	93.3	80.0			0.63	0.05–7.72
Widowed	2.7	17.5			0.06*	0.00–1.24
Education						
No formal education	57.3	80.0			1.00	
Primary	28.0	15.0			1.68	0.56–5.06
Secondary +	14.7	5.0			5.07*	0.90–28.37
Religion						
Traditionalist	7.3	12.5				
Catholic	36.0	20.0				
Protestant	5.3	7.5				
Moslem	51.3	60.0				
Model diagnostics						
No. of observations			190		190	
Likelihood ratio (*df*) χ^2^; *p*-value			26.26(6); 0.0002		47.24(14); 0.0000	
Pseudo-*R* ^2^			0.1343		0.2415	
H–L test (*df*) χ ^2^; *p*–value			1.86 (8); 0.985		5.62 (8); 0.6895	

* *p*<0.1

** *p*<0.05

*** *p*<0.01.

Data from the key informant interviews and stakeholder discussions were tabulated and manually analysed. Content analysis of the in-depth interviews and project management discussion transcripts (and notes) was carried out.

## Results

### Background characteristics

Two hundred and fifty households were interviewed out of the 260 sampled, giving a 96% response rate. The household respondents were mostly illiterates (67%), urban residents (57%), farmers (63%), and Muslims (57%). The ages of the respondents ranged from 18 to 66 years, with an average age of 40 years. The average household size was eight persons. Nearly two-thirds (64%) of the population owned radio sets and one-fifth (20%) owned a television set (see Table 1). Television sets owners lived in Nouna Township.

### Awareness, knowledge and enrolment

The CHI campaign reached 77% (193) of the household respondents and 23.2% enrolled in the scheme. The relationship between awareness and enrolment was observed to be statistically significant (*p*<0.001). All 22 community leaders heard about the CHI initiative through their participation in the advocacy seminar, radio discussions, congregational announcements in mosques and churches, and at the formal launch of the scheme.

Of those who were exposed to the campaign, 79% (152) had adequate knowledge about the scheme, but just a little more than a third (35.3%) enrolled in the scheme. Thus, a significant proportion of the respondents with adequate knowledge did not enrol (*p*<0.001). It was also observed that only 7.5% of those without adequate knowledge enrolled. A high proportion, 66% (127) of households who heard about the scheme also shared information with relatives and friends. Fifty-three percent of those who had indirect exposure to the campaign messages also had adequate knowledge about the scheme. All of the community leaders clearly understood the CHI scheme concept, as indicated below:

CHI means everybody contributes, whether sick or not, so that we all help each other or share the cost of health care, and in that way even the poor who cannot afford it can also receive care. [Religious leader]

A positive association was found between educational status and level of knowledge about the CHI scheme (*p*<0.026), and between educational status and enrolment (*p*<0.001). A much higher proportion of rural residents (84.7%) had adequate knowledge about the scheme than their urban counterparts (74.3%).

Nearly, three-quarters (73.9%) of the respondents reported that their community heads participated in the CHI campaign. More community heads in rural areas (66%) than their urban counterparts (47%) participated in the campaign. Household CHI awareness, knowledge, and enrolment were higher in communities where the heads (*Délégates*) participated in the campaign, compared with where they did not. The difference was observed to be statistically significant in household awareness [*X* ^2^ (1, *N*=206) = 20.258, *p*=0.001], adequacy of CHI knowledge [*X* ^2^ (2, *N*=188) = 29.353, *p*<0.001], and enrolment (35.3% vs. 15.6%, *p*<0.0001).

Community leaders and CHI Management asserted that the involvement of community heads and religious leaders was vital in the scheme promotion strategy. Of particular importance was the sense of ownership resulting from active engagement of community heads in the campaign. Decision makers were of the opinion that the project did engage them appropriately:

The CHI involved all people and placed mobilization and education in the hand of the community leaders [Community Head]

The CBI initiative belongs to us –Nouna District and the Research Centre are providing technical support; they have shown a lot of respect and concern. [Steering Committee member]

The in-depth interview respondents were impressed with the campaign strategy that combined mass, interpersonal, and folk media. For example, they described radio as a credible national tool for promoting developmental programmes, including health initiatives and considered it appropriate for the CHI promotion. Of particular interest was the need to intensify radio discussions, the channel through which most of the population heard about the scheme, which in their opinion, would help the campaign to gain prominence and facilitate the achievement of better communication outcomes.

The Radio discussion by CEDICOM gave a clear understanding of the CHI scheme and it built trust among audience about the initiative. It also reaches the entire Kossi province, which means that people can hear about the CHI initiative, and the discussion in Dioula was very useful; it makes everyone understand. [Community Head]

Although information vans and posters were used extensively, they did not appeal much to the decision makers. The majority of the leaders considered these as awareness creation tools. The mobile van, in particular, was perceived as a tool for propaganda used by politicians and therefore not ideal for improving people's understanding on an important issue such as the CHI scheme. A head of an NGO said:

… it moves fast and makes much noise so you cannot focus on the messages, all you hear is allo, allo. [Head of an NGO/Community]

Community leaders also noted the limited use of traditional folk media, which were widely known by the population and likely to be more effective than radio. They suggested the use of traditional channels and sources that promote real engagement with the communities such as the COGES, *Griots*, and community drama performances with follow-up discussions among small groups of audiences. These, in their view, would not only promote understanding but also motivate them to take action.

The Griots and Animateurs help put the information at the door-steps of our rural people and they are the ones people contact for further explanation on issues; they are good for promoting every initiative. [Religious Leader]

Of particular concern was the perception that the campaign focused heavily on rural areas to the disadvantage of urban residents. The majority of community leaders were of the view that urban sensitisation should receive the same attention as rural areas, since rural residents tend to rely on their relatives in urban centres to act on information.

The CHI team did not realize that villagers have relatives in Nouna and tend to confirm their understanding of issues and decision to act on information based on our recommendations. [Association Leader]

### Campaign effect on knowledge

Exposure to multiple channels was the only significant and positive determinant of respondents’ knowledge [*p*≤0.05] (see Model 1 in Table 2). The rest of the IEC variables were not significant even at *p*≤ 0.10. Though not significant, individuals who had frequent access to CHI messages from the CHI team and whose leaders participated in the promotion were 2.4 and 1.4 times more likely to exhibit adequate knowledge than those who did not benefit from such situations. The effect of high exposure to channels on respondents’ knowledge was significantly enhanced in the presence of socio-demographic variables [*p*<0.05] (see Model 2 in Table 2). Of particular importance in influencing knowledge was secondary education. Respondents with secondary or higher education were five times more likely to possess adequate knowledge on the CHI scheme than those with no formal education. Interestingly, residents in urban areas were 2.4 times less likely to have knowledge of the CHI scheme.

### Campaign effect on enrolment

It was observed that intensity of exposure was the only significant IEC predictor of CHI enrolment [*p*≤ 0.01] (Model 1 in Table 3). Respondents with access to two or more channels were about 7.8 and 6.7 times more likely to enrol than those who had no access. This meant that the odds of enrolment increased with the number of IEC channels that individual households had access to. The rest of the IEC variables were found not to be significant even at the 10% level of significance. In Model 2, the results showed that intensity of exposure to campaign channels, community participation, respondents aged between 36 and 54 years and having secondary or higher education were positive and significant determinants of enrolment. Ownership of radio alone or radio and television sets were also found to be significant determinants of enrolment in the scheme.

**Table 3:** Multivariate logistic regression on determinants of enrolment in CHI

			Regression results			
			
	Enrolment status		Model 1		Model 2	
			
Variables	Not enrolled (*n*=193) %	Enrolled (*n*=57) %	OR	95% CI	OR	95% CI
IEC variables						
Exposure to channels						
None	44.0	5.3	1.00		1.00	
One	32.6	47.4	7.84***	2.15–28.55	6.04**	1.51–24.25
Two or more	23.3	47.4	6.69***	1.59–28.12	6.27**	1.30–30.32
Frequency of info. from CHI team						
Less frequent	87.0	63.2	1.00		1.00	
Frequent	13.0	36.8	1.73	0.80–3.72	1.18	0.48–2.87
Frequency of info. from mass media						
Less frequent	80.3	49.1	1.00		1.00	
Frequent	19.7	50.9	1.79	0.84–3.82	1.82	0.74–4.46
Community participation						
No participation	61.1	28.1	1.00		1.00	
Participation	38.9	71.9	1.55	0.73–3.30	2.32*	0.89–6.04
Socio-demographic characteristics						
Sex						
Female	22.4	10.5			1.00	
Male	88.6	89.5			1.63	0.49–5.42
Age in years						
< 36	26.4	17.5			1.00	
36–54	64.2	77.2			2.47*	0.94–6.53
≥ 55	9.3	5.3			2.62	0.40–17.33
Household size						
< 5	22.8	12.3			1.00	
5–9	51.8	61.4			1.75	0.55–5.62
≥ 10	25.4	26.3			1.14	0.29–4.54
Residence						
Urban	57.0	57.9			1.00	
Rural	43.0	42.1			0.99	0.38–2.58
Marital status						
Never married	4.7	3.5			1.00	
Married	86.5	93.0			0.43	0.04–4.25
Widowed	8.8	3.5			0.32	0.02–5.87
Religion						
Traditionalist	8.3	7.0			1.00	
Catholic	28.0	36.8			0.82	0.19–3.65
Protestant	5.7	3.5			0.41	0.04–4.04
Moslem	58.0	52.6			0.89	0.18–4.35
Education						
No formal education	75.6	38.6			1.00	
Primary	16.6	35.1			1.87	0.73–4.79
Secondary +	7.8	26.3			6.11***	1.57–23.70
Ethnicity						
Others	24.9	17.5			1.00	
Bwaba	23.8	33.3			2.13	0.50–9.19
Darfing	26.4	29.8			2.76	0.77–9.81
Mossi	14.0	8.8			1.99	0.43–9.24
Samagho	10.9	10.5			3.21	0.71–14.45
Ownership of assets						
None	40.4	15.8			1.00	
Radio	44.0	47.4			2.81*	1.00–7.93
Radio and television set	15.5	36.8			4.57**	1.18–17.61
Model diagnostics						
No. of observations			250		250	
Likelihood ratio (*df*) χ^2^; *p*–value			44.79(5): 0.0000		78.61(24); 0.000	
Pseudo-*R* ^2^			0.1669		0.2928	
H–L test (*df*) χ^2^ ; *p*-value			1.96 (5); 0.8551		3.24(8); 0.9185	

* *p*<0.1

** *p*<0.05

*** *p*<0.01.

## Discussion

Our findings show that IEC campaigns can effectively enhance a household's knowledge about the CHI initiative, and to a lesser extent, promote household uptake of the scheme. The study identified two IEC variables as the most important determinants of household knowledge and enrolment in the scheme: exposure to multiple channels and participation of community heads. Having formal education, being between 36 and 54 years of age and having ownership of a radio and television were also identified as other socio-demographic factors that enhanced enrolment in the scheme.

As expected, the frequent exposure and access to clear, consistent messages from credible channels and sources did not only raise household awareness about the scheme, but also improved knowledge about the CHI scheme. This finding is consistent with previous evaluation studies on IEC campaign promotion of community-based health initiatives (11, 22, 25, 28, 34). The synergistic effect of multiple media (interpersonal mass and folk media channels) provided ample opportunities for the target communities to learn and improve their knowledge about the CHI scheme. For example, radio provided opportunities to households in all the target communities to receive the CHI campaign messages simultaneously while the community and group discussions, together with the continuous broadcast through the information van, created a synergetic effect towards improving households’ knowledge about the CHI scheme.

Community leaders and personal field observations also confirmed the reinforcing and complementary nature of the messages from the multiple media sources. Of particular importance was the observation that respondents who received frequent messages from more than two channels had a better understanding of the scheme than others. This finding corroborates conclusions drawn by studies in family health and HIV/AIDS in Uganda and some West African countries, which suggest that a ‘dose-response effect’ is achieved when people receive messages from multiple communication channels (34, 35).

Among the channels, radio had the greatest reach and credibility in Burkina Faso, and should continue to receive priority in CHI promotion activities. The indirect channel (family, friends, relatives and social networks) was also a major source of campaign message for a reasonable proportion of household respondents. These conversations subsequently translated into actual knowledge improvement, which suggests that actively leveraging social networks to directly spread campaign messages might be a good strategy for CHI promotion.

Contrary to expectations, our findings showed that residing in the urban setting did not increase the odds of having knowledge of the scheme. As indicated by community leaders, rural inhabitants tended to validate information from their relatives in urban areas. This means that a clear understanding of the CHI concept by urban dwellers would be paramount to decision making and adoption of the initiative by many rural residents. Thus, it will be valuable for future campaigns to diffuse messages fairly across settings to achieve a modest impact.

Exposure intensity and community participation were the only significant IEC determinant of household enrolment. We observed that the level of households’ exposure to multiple channels remained a predictor of household enrolment in the presence of socio-demographic variables. This implies that if households have access to information from different channels, they are likely to enrol in the CHI scheme.

Among the socio-demographic factors, our study found formal education attainment, specifically secondary and higher education, the matured and active working age group between 36 and 54 years as a positive influence on the likelihood of enrolling in the scheme, which is consistent with earlier studies (8). Consistent with earlier study in Rwanda, radio and television ownership were major determinants of enrolment. It is likely that such households may have regular exposure and access to CHI campaign messages and are wealthy enough to afford the premium payment (36).

Our analysis points to a positive association between community participation and households’ enrolment in the scheme. Our field observations also revealed relatively high CHI enrolment rates in communities with active leadership participation. This confirms views shared by community leaders during the in-depth interviews on their ability to influence and motivate their constituencies to embrace initiatives. This also reinforces the conclusion drawn by health promotion and communication experts on the tremendous effect of community leadership's participation in influencing beneficiary action on health initiatives; as leaders are often able to facilitate the creation of demand for the initiatives (24–26, 37, 38). Our findings therefore suggest the need to engage these valuable sources in CHI campaigns to effectively influence community attitudes and behaviours in the adoption of health the CHI initiatives.

The positive association between education attainment and enrolment may partly be due to improved purchasing power – most educated respondents were also income earners and more likely to afford payment of insurance premiums. This also underscores the importance of an IEC campaign to promote the CHI scheme especially in predominantly illiterate communities. Such campaigns should go beyond providing information about the scheme concept and benefits to targeting appropriate social and behavioural variables to make an impact.

Our study found a positive association between the 35 and 54 years age group and enrolment. This may be due to their sense of maturity and the importance that they place on better health status for their families, which translates to value for the scheme. Champions should therefore be identified from this group to promote the scheme.

Our study did not, however, analyse the interactions between the exposure and the other campaign variables. It also did not differentiate in the analysis between the effects of individual mass media channels on outcomes. Future studies should therefore examine the interactions between CHI campaign components, as well as the effectiveness of selected individual campaign channels rather than treating them in groups.

Our findings show that even though household knowledge about CHI was high, its effect on enrolment was minimal. A significant proportion of the household respondents had a clear understanding of the CHI scheme and had expressed a willingness to contribute to premium payment (39–41) but failed to enrol at the end of the campaign. This finding confirms the widely accepted hypothesis that knowledge acquisition is important, and it does not necessarily translate into adoption on its own (42, 43).

For some, it may be a mere *let us wait and see how it works* attitude, which is grounded in behaviour change models (44). It also means that the relationship between household knowledge acquisition and enrolment is not linear, and can be mediated by a number of factors. As found by other studies conducted in this setting and elsewhere, socio-economic, cultural, demographic, and health system factors influence decisions to enrol in a CHI scheme (5, 9, 10, 45–47). Factors such as perceived poor quality of care; lack of trust related to previous negative experiences with collective financial arrangements; and providers’ resistance have been cited to affect the decision to enrol in CHI. The interplay between knowledge and enrolment warrants further studies. IEC evaluation should broaden its focus beyond knowledge as enrolment can be attained through other linkages. The CHI campaign should focus on improving understanding of the concept and principles as well as predictors of CHI compliance, for example, counter scepticisms about the viability of the scheme, benefits, and healthcare quality (5, 45, 47).

### Limitations

Our conclusion in this study should be taken with caution due to potential limitations. Our study design was non-experimental rather than experimental or quasi-experimental. The latter design could not be applied due to the nature of the campaign, timing, and limited funds for the study.

The in-depth interviews were biased towards the literate population who happened to be in key positions in the district, including the village Délégates. Also, there were apparent intercorrelations among the IEC variables such that intensity of exposure was the only significant variable in the multivariate analysis. These limitations notwithstanding, triangulating data from the different sources ensured the credibility of our findings (48, 49).

## Conclusion

IEC played a crucial role in improving household understanding and adoption of the CHI scheme and is worth inclusion in scheme design, implementation and evaluation. The most crucial and effective campaign factors identified in this study were intensity of households’ exposure to multiple media channels and community leaders’ participation. High exposure to both mass and interpersonal channels had a strong effect on CHI knowledge and enrolment. Community leaders appear to have influence on enrolment, and their inclusion in campaign design and evaluation should be emphasised to enhance the impact on CHI programmes. Strategically planned and inclusion of radio discussions as well as traditional folk media that integrates social values and norms in an enthusiastic and educative manner could influence household enrolment in the scheme and should be considered in future campaigns.

Education attainment was the only influential socio-demographic determinant of both knowledge and enrolment among household heads. The relatively low effects of the IEC campaign on CHI enrolment are indicative of other important IEC mediating factors, which should be taken into account in future CHI campaign evaluations.

It is worth noting that our study examined only two of the multiple links between the CHI campaign promotion and household CHI enrolment. Future studies need to build on ours by focusing on the important enabling factors in the knowledge–enrolment path.
